# Survival Analysis of Male Patients with Brain Metastases at Initial Breast Cancer Diagnosis over the Last Decade

**DOI:** 10.3390/medsci12010015

**Published:** 2024-03-10

**Authors:** Jorge Avila, Julieta Leone, Carlos T. Vallejo, José P. Leone

**Affiliations:** 1 Department of Medicine, St Elizabeth’s Medical Center, Tufts University School of Medicine, Boston, MA 02135, USA; jorge.avilazambrano@steward.org; 2 Grupo Oncológico Cooperativo Del Sur (GOCS), Neuquén Q8300HDH, Argentina; 3 Medical Oncology, Dana-Farber Cancer Institute, Susan F. Smith Center for Women’s Cancer, Harvard Medical School, Boston, MA 02215, USA

**Keywords:** male breast cancer, brain metastases, overall survival

## Abstract

**Simple Summary:**

Breast cancer in men is a very rare disease, representing less than 1% of all breast cancer diagnoses. An important percentage of patients with breast cancer will develop brain metastases during the course of their disease. There is very limited data about male patients with breast cancer and brain metastases. Our study evaluated a national database that included 22 patients with brain metastases at initial breast cancer diagnosis and found that tumor subtype, marital status, tumor grade, and other sociodemographic factors affect survival in this population. Further studies including new drugs are recommended to fully evaluate the response to novel treatments for this disease.

**Abstract:**

Breast cancer in men represents approximately 1% of all breast cancer diagnoses. Among all patients with breast cancer, approximately 30% will develop brain metastases. Over the past decade, there have been multiple advances in the treatment of metastatic breast cancer; however, long-term outcomes of this presentation in male patients are lacking. We evaluated male patients with de novo stage IV breast cancer using the Surveillance, Epidemiology and End Results (SEER) database from 2010 to 2019. Overall survival (OS) was estimated using the Kaplan–Meier method and differences between groups were compared using log rank tests. In total, 22 male patients with brain metastases at initial breast cancer diagnosis were included. Patients with HR-positive/HER2-negative tumors had the longest OS (median 13 months). Factors associated with shorter overall survival were advanced age, unmarried marital status, lower household income, and grade III disease, among others. Brain metastases remains an unmet medical need for patients with breast cancer; the development of new drugs may provide an improvement in overall survival for male patients in the future.

## 1. Introduction

Breast cancer in men represents approximately 1% of all breast cancer diagnoses [1]. For men in the United States, the lifetime risk of getting breast cancer is about 1 in 833, although there is global variation of this disease with higher incidence in countries like Israel, The Philippines, and France [2]. The incidence of breast cancer in men appears to be rising, as data suggest that the incidence has increased by 7.2% to 10.3% in the last decade [3].

Different risks factors have been associated to developing breast cancer in men including obesity, family history of breast cancer, black race, and exposure of the breast to ionizing radiation, among others [4]; however, the two most significant factors that have been described are carrying a diagnosis of Klinefelter’s syndrome and having a predisposition germline genetic mutation (BRCA2, BRCA1, CHEK2, PALB2) [5,6,7]. Using DNA testing from 1355 men with breast cancer, Moelans et al. demonstrated that 15% of the patients from their study presented at least one of these genetic characteristics [8].

Brain metastases is a challenging presentation of breast cancer, often associated with poor prognosis, as the blood–brain barrier prevents the delivery of many drugs to the central nervous system leading to a decline in functional status and quality of life [9,10].

Around 30% of all patients with breast cancer will develop brain metastases; however, given that male breast cancer is an uncommon disease, the incidence of brain metastases from male breast cancer is even rarer [11]. Nonetheless, more than 40% of male breast cancer patients present with advance stage at initial diagnoses, including larger tumor size and higher risk of lymph node involvement, most likely due to the absence of routine screening, delayed identification of the disease, and anatomical differences predisposing to local invasion [12,13].

Studies have demonstrated that breast cancer in men is more often Hormone Receptor (HR) positive when compared to female breast cancer, and its prevalence is similar to that in postmenopausal women, which indicates that breast cancer in men is usually responsive to anti-hormonal therapies like tamoxifen [14]. Cardoso et al. showed that, in a population-based study including approximately 1500 male breast cancer patients, 99% of them were estrogen receptor positive, 97% were androgen receptor positive, and 81% were progesterone receptor positive [15].

The risk of brain metastases is higher in patients with HR-negative, human epidermal growth factor receptor 2 (HER2)-positive, or triple negative tumors [16]. It has been shown that tumor subtype also plays a role in terms of median time interval from primary diagnosis to the development of brain metastases, and studies have demonstrated shorter intervals for triple negative and HER2-positive tumors, and longer intervals for estrogen receptor-positive tumors [17].

Median overall survival (OS) in patients with breast cancer brain metastases ranges from 2 to 25.3 months [18,19,20]; however, some studies have demonstrated worse survival for men with breast cancer when compared to women, while others have shown similar survival between genders [21,22].

In the U.S., approximately 5% of patients with breast cancer will present with de novo metastatic involvement [23], and among them, 7.26% will present with brain metastases at initial breast cancer diagnosis [24]. However, due to the sparsity of cases, epidemiologic data of this presentation on male breast cancer is lacking.

The Surveillance, Epidemiology and End Results (SEER) database is a federally funded, publicly available, cancer reporting system that represents a collaboration between the United States Center of Disease Control and Prevention, the National Cancer Institute and regional and state cancer registries [25]. The SEER database includes all patients regardless of their performance status or organ function and includes data about the primary tumor site, tumor morphology, stage at diagnosis, and first course of treatment [26].

There have been significant advances in the treatment of metastatic breast cancer over the past decade (2010–2019); however, outcomes of breast cancer brain metastases are lacking, especially for men. Therefore, the goal of this study was to identify clinical and sociodemographic factors associated with OS in male patients with brain metastases at initial breast cancer diagnosis.

## 2. Materials and Methods

We evaluated male patients with de novo stage IV breast cancer using the SEER database from 2010 to 2019. Patients with other primary malignancies were excluded.

The SEER database includes data on cancer incidence, survival, and treatment for approximately 30% of the United States population. It also collects data on patient demographics, primary tumor site, stage at diagnosis, and tumor morphology. SEER has collected information about sites of metastases at initial diagnosis since 2010, which is the year we are using as the starting point for this investigation.

The variables included in this study were race, age at diagnosis, year of diagnosis tumor histology, tumor subtype, and tumor grade, as well as additional sites of metastasis. We also collected data for treatment including chemotherapy, radiation therapy, and surgery, as well as sociodemographic factors like marital status at diagnosis, and annual income.

The race variable was divided into three subcategories: <50 years old, 50–64, and >64 years of age. Race was subdivided into the following categories: Non-Hispanic White, Non-Hispanic Black, Non-Hispanic Pacific Islander, and Hispanic.

The variable tumor subtype was divided depending on the presence or absence of different receptors into the following subcategories: HR-positive/HER2-negative, HR-positive/HER2-positive, HR-negative/HER2-positive, triple negative, and unknown. Histology was also subcategorized into 4 groups: lobular, ductal, mixed lobular and ductal, and other. Tumor grade was divided into 3 categories: I, II, and III/IV, with grade III and IV merged into one variable. Extracranial metastases at time of diagnosis included bone, liver, lung, distant lymph node, and other.

Variables for treatment included: chemotherapy, radiation therapy, and surgery. The variable surgery was subdivided into Yes and No, depending on whether or not the patient had had any surgical intervention, including lumpectomy or mastectomy. The radiation and chemotherapy variables were divided into Yes and No/Unknown.

The variable marital status at diagnosis was subdivided into 4 categories: single, married, other (including divorced, separated, and widowed), and unknown.

Patient characteristics were tabulated by frequencies and proportions for all stage IV breast cancer patients. OS probabilities were estimated using the Kaplan–Meier method and the log rank test was used to compare the differences between groups. All *p* values < 0.05 were considered statistically significant. Statistical analyses were performed using STATA version 12 and SPSS version 26. Institutional review board approval was not necessary as no identifiable data were utilized in our study.

## 3. Results

A total of 313 male patients with de novo metastatic breast cancer were identified from 2010 to 2019; and among them, 22 (7.03%) presented with brain metastases at initial breast cancer diagnosis. Within this group of patients, the median age was 55 years (interquartile range 49–69 years).

In terms of race, 15 patients (68.2%) were Non-Hispanic white, and 3 patients (13.6%) were Non-Hispanic black. In terms of breast cancer tumor subtype, 9 patients (40.9%) had HR-positive/HER2-negative tumors, 5 (22.7%) had HR-positive/HER2-positive tumors, 1 (4.5%) had HR-negative/HER2-positive tumor, and 3 (13.6%) had triple negative tumors. The majority of the patients (50%) had grade III/IV tumors while only 5 (22.7%) patients presented grade II tumors and no patients (0%) had grade I tumors. In terms of histology, all patients (100%) presented tumors with ductal histology. In addition to brain metastases, 16 patients (72.7%) had additional bone metastases, 4 (18.2%) had additional liver metastases, 12 (54.5%) had additional lung metastases, and 1 (4.5%) had distant lymph node metastases.

Sociodemographic characteristics were also evaluated in our population. Most of the patients included in our study (36.4%) had a median household income higher or equal to USD 75,000, meanwhile only 4.5% had an income between USD 35,000 and 44,999. In total, 13 (59.1%) of the patients included in our study lived in metropolitan areas with a population of ≥1 million habitants, while only 2 (9.1%) lived in nonmetropolitan areas not adjacent to metropolitan areas. In terms of marital status, 12 patients (54.5%) were married and 6 (27.3%) were single.

In terms of cancer treatments, 4 patients (18.2%) underwent surgery (mastectomy or lumpectomy), while the majority of the patients (81.8%) did not undergo any surgical procedure; 13 patients (58.1%) underwent radiation therapy and 16 (72.7%) received chemotherapy. Table 1 shows the complete distribution of patient characteristics.

In the cohort of patients with Stage IV disease without brain metastases (*n* = 291), most of the patients were older than 64 years of age (49,8%), while only 37 (12.7%) were younger than 50 years old. A total of 184 patients (63.2%) were Non-Hispanic white, 59 (20.3%) were Non-Hispanic Black, and 23 (7.9%) were Hispanic. The majority of the patients (60.8%) had HR-positive/HER2-negative tumors, while only 1 (0.3%) presented with HR-negative/HER2-positive disease. A total of 100 patients (34.4%) had grade III/IV tumors, and 13 (4.5%) had grade I tumors. In terms of sociodemographic factors. Most of the patients within this cohort (51.2%) were married and 67 (23%) were single. Most of the patients (34.4%) had a median household income higher or equal to USD 75,000, meanwhile only 8.2% had an income between USD 35,000 and 44,999. A total of 169 (58.1%) patients from this cohort lived in metropolitan areas with a population ≥1 million habitants, while only 15 (5.2%) lived in nonmetropolitan areas not adjacent to metropolitan areas. In terms of cause of death, 154 patients (52.9%) died from breast cancer, meanwhile 16 (5.5%) died from other causes.

**Figure 1:**
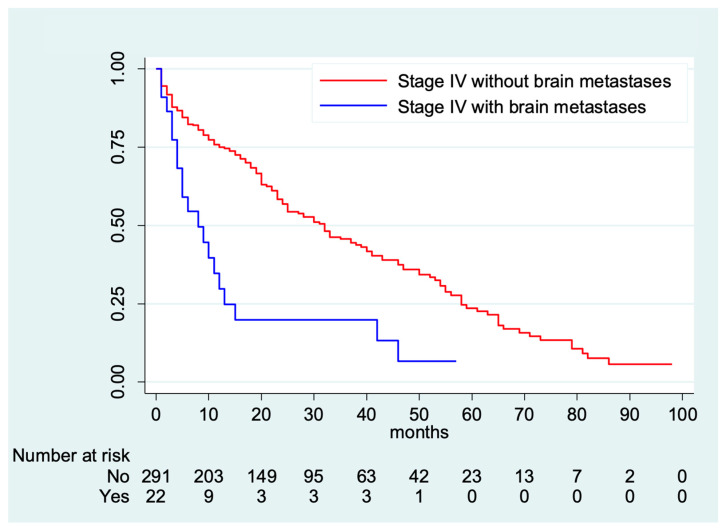
Overall survival for men with stage IV at initial breast cancer diagnosis.

The median follow-up of the entire cohort of stage IV breast cancer patients (*n* = 313) was 45 months (Interquartile range 21–79 months). There were significant differences in OS according to the presence or absence of brain metastases at initial diagnosis. Patients without brain metastases (*n* = 291) had a median OS of 32 months (95% CI 25–39 months); whereas men who presented with brain metastases at initial breast cancer diagnosis (*n* = 22) had a median OS of 8 months (95% CI 4–12 months; *p* < 0.001) (Figure 1). In the brain metastases cohort, there were 19 (86.4%) total deaths reported, of which 18 were due to breast cancer.

**Figure 2:**
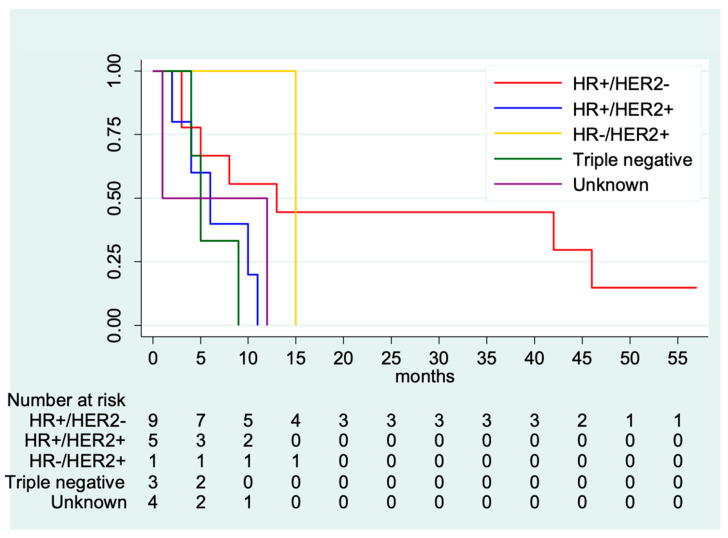
Overall survival divided by tumor subtype. Abbreviations: HR, hormone receptor; HER, human epidermal growth factor receptor.

In the brain metastases cohort, univariate analysis of OS according to clinical characteristics showed that patients who were younger (<50 years of age) experienced numerically longer OS (median OS: 9 months; 95% CI 1–42 months) compared with those who were >64 years of age, who had a median OS of 4 months (95% CI 1—not estimable; *p* = 0.53). Non-Hispanic white patients had a median OS of 9 months (95% CI 3–12 months), Hispanic patients had a median OS of 8 months (95% CI 8—not estimable), Non-Hispanic Black patients had a median OS of 5 months (95% CI 1—not estimable), and Non-Hispanic Asian or Pacific Islander had a median OS of 1 month (95% CI 1—not estimable; *p* = 0.59). OS according to tumor subtype showed that patients with brain metastases from triple negative breast cancer experienced the shortest survival (median OS: 5 months; 95% CI: 4—not estimable), whereas patients with HR-positive and HER2-positive presented a median OS of 6 months (95% CI 2—not estimable) and those with HR-positive/HER2-negative had a median OS of 13 months (95% CI: 3–46 months; *p* = 0.19). (Figure 2). Among other sociodemographic factors, married patients had a numerically longer OS when compared to their single counterparts (8 vs. 5 months; *p* = 0.25). In terms of extracranial metastases, patients with additional liver metastases had a significantly lower OS when compared to those who did not (2 vs. 8 months, respectively; *p* = 0.04). Patients who did not receive chemotherapy had a significantly lower OS when compared to those who did (3 vs. 10 months, respectively; *p* = 0.004). Table 2 shows OS based on clinical and sociodemographic characteristics. 

## 4. Discussion

Factors associated with OS and prognosis in breast cancer are well studied in female patients; however, this is not the case for males, who account for less than 1% of all breast cancer diagnoses. There are differences in the evaluation of data for metastatic breast cancer in male when compared to female patients.

To our knowledge, this is the largest study to analyze survival in male patients with brain metastases at initial breast cancer diagnosis, which provides relevant data about an uncommon disease. The median OS of 8 months in our population is similar to the one presented in other investigations in the previous years [18,24,27]; however, as mentioned before, our article is the first one to solely focus on male patients.

The present study showed that, in agreement with prior investigations [15,28], the majority of male patients with breast cancer have HR-positive/HER2-negative tumors, meanwhile HR-negative/HER2-positive remains to be the least common subtype among men.

Difference in survival for male patients with breast cancer has presented different data. Some results have shown that patients with HER2-positive tumors have significantly worse OS when compared to those with HER2-negative disease independent from HR status [29], while others have found no significant differences between tumor subtypes [30]. Despite the poor OS, 55% of patients who had HR-positive/HER2-negative tumors were alive at 1 year, and 44% were alive at 2 years. On the other hand, no patient with triple negative breast cancer survived longer than 1 year. However, no significant differences were found between OS based on tumor subtype. This could be explained by the small number of patients included in our study.

Among male patients who presented with brain metastases at initial breast cancer diagnosis, poor OS was associated with advanced age, triple negative subtype, no primary surgery, no chemotherapy, and additional visceral metastases. These results are consistent with recent reports from SEER [24,31] and highlight the relevance of patient characteristics, tumor factors, and sociodemographic factors in the prognosis of patients with breast cancer brain metastases.

Social support remains an important factor for survival in cancer patients overall. Our study demonstrated that married patients had a numerically longer OS when compared to their unmarried counterparts (8 vs. 5 months). Different studies have shown that unmarried patients are at significantly higher risk of presenting with metastatic disease, undertreatment, and death resulting from their cancer [32]. For breast cancer, Zhu and Lei evaluated the SEER database and found that unmarried patients had a 15.5% increased risk of breast cancer-specific mortality and a 19% increased risk of overall mortality when compared to married patients with metastatic disease [33].

Various studies have shown differences in survival among patients with metastatic breast cancer based on their race, demonstrating that Non-Hispanic black patients can be as high as 57% more likely to die from this disease than Non-Hispanic white [34,35]. Our data showed that these disparities are also prevalent in male patients with breast cancer, as Non-Hispanic white patients presented a numerically longer OS when compared to Non-Hispanic Black patients (9 vs. 5 months) in our study.

Patients with breast cancer brain metastases continue to have a worse overall survival when compared to those without brain metastases. Previous data have shown that patients with bone metastases seem to have the best OS among the extracranial metastases group and a significantly longer OS compared to the brain metastases group (36 vs. 8 months) [36].

In terms of heterogeneity and spatial distribution of brain metastases based on breast cancer subtype, Kyeong et al. reviewed images from 100 patients with breast cancer brain metastases and reported that the number of brain metastases did not differ significantly by tumor subtype. They also found that brain metastases from triple negative breast cancer occurred more often in the frontal lobe, limbic region, and parietal lobe when compared dominantly in the occipital love and cerebellum [37]. Unfortunately, SEER does not provide data about specific location or number of brain metastases; therefore, we cannot compare this information with our findings.

Unfortunately, male breast cancer can sometimes be identified late, with lymph node involvement and advanced staging by the time of diagnosis, hence its worse prognosis [38]. Different studies have shown that the incidence of distant metastases is higher in male breast cancer, when compared to female breast cancer [39,40]. This seems to be related to the lack of knowledge about this disease in the general population. One study including 411 patients performed by Altiner et al. demonstrated that more than half of the participants (61.1%) were not aware of the possibility of this disease in men [41]. Raising awareness of this uncommon disease will enable men to be diagnosed earlier and possibly increase their survival time. One population-based study including 2665 men diagnosed with breast cancer from Sweden, Denmark, Finland, Geneva, Norway, and Singapore showed that male patients had a worse 5 year relative survival ratio when compared to women, although, after adjusting for age, year of diagnosis, stage and treatment, male patients had a significantly better relative survival from breast cancer than female patients [42]. However, other studies using the SEER database have demonstrated that there is no difference in prognosis between metastatic breast cancer in men and women. Outcomes for female breast cancer patients with brain metastases have been reported in prior SEER studies and have shown a median OS of 10 months [43]. It has also been shown that when compared to breast cancer in women, male breast cancer has a higher proportion of presenting with simultaneous bone and lung metastases [40].

SEER does not provide any specific information about chemotherapy regimens and therefore we cannot evaluate the prognostic impact of individual treatments in our population. However, given that most of our patients had HR-positive tumors, we can assume that the majority of them received endocrine therapy. Guidelines mention that men with HR-positive breast cancer who are candidates for adjuvant endocrine therapy should be offered tamoxifen. Nonetheless, those who have a contraindication to tamoxifen may be offered a gonadotropin-releasing hormone (GnRH) agonist/antagonist and an aromatase inhibitor [7].

Other possible options for treatment could be chemotherapy including anthracycline-containing and anthracycline-free regimens. Chemotherapy has been shown to have anti-tumor activity in metastatic breast cancer in male patients previously treated with endocrine therapy [44].

Future studies may be able to compare results from the next decade to the ones presented in our analysis to further evaluate the long-term effects of new treatments like immunotherapy, antibody drug conjugates (ADCs), and Poly (ADP-ribose) polymerase (PARP) inhibitors in patients with breast cancer brain metastases.

Given that approximately 20% of male breast cancer patients discontinue tamoxifen within the first 1 to 2 years of starting treatment [45], it is possible that our results are affected by a lack of compliance to treatment; however, SEER does not provide data about this. It is important to note that some studies suggest that when compared to women, men are less likely to report side effects, but more likely to discontinue treatment earlier [46]. Different studies have shown that the most common side effects in male patients with breast cancer are fatigue, fear of recurrence, swollen arm, difficulty in arm movement, and in some cases changes in erectile function, orgasmic function, and sexual desire. It is also important to remember that common side effects like hair loss are coped with differently by men and women, as men can have hair loss in the chest as a result of radiation, which can lead to significant distress and impact the experience of men during cancer treatment, while most women do not experience hair loss in this area [47].

Our study had some limitations. Given the small number of patients included in it, we do not have enough power to detect significant differences within the subgroup of patients with brain metastases. Second, SEER only reports data for the first course of therapy; therefore, we are not able to evaluate the impact of therapies in patients who were catalogued as not receiving radiation, chemotherapy, or surgery, but may have undergone these afterwards. Another limitation is that SEER does not report information about number of brain metastases and Karnofsky performance status, which have been shown to be important prognostic factors in multiple studies with brain metastases [48]. In addition, we cannot assess the impact of the implementation of new drugs like trastuzumab deruxtecan, tucatinib, or sacituzumab govitecan [49,50,51], which have been shown to have a positive impact on survival in the general population, as these agents were approved after 2019. Finally, SEER does not include data for side effects and possible factors leading to discontinuation of treatment.

Future research will help us evaluate if the addition of new drugs and targeted therapies will have a significant impact on the prognosis of male patients with brain metastases at initial breast cancer diagnosis. It would also be important to investigate the effect of specific therapies in male patients exclusively as treatments and outcomes for male breast cancer are often extrapolated from studies conducted in women. The lack of data in this population is reflected by the fact that of 131 randomized breast cancer clinical trials, male patients represented only 0.09% of the total study population, and only 27 trials included male patients [14].

## 5. Conclusions

Brain metastases remains an unmet clinical need for all breast cancer patients, and this seems to include male patients with breast cancer. Over the last decade, the OS for male patients with brain metastases at initial breast cancer diagnosis remains poor, specially for those with the triple negative tumor subtype. However, there are longer term survival rates in patients with HR-positive/HER2-negative tumors. OS also varied depending on age, extracranial metastases, and sociodemographic factors.

## Figures and Tables

**Table 1:** Demographic and clinical characteristics of the patients.

		*N*	%
**All patients**		22	100%
Age	<50	7	31.8%
	50–64	7	31.8%
	>64	8	36.4%
Race	Non-Hispanic White	15	68.2%
	Non-Hispanic Black	3	13.6%
	NHAPI	2	9.1%
	Hispanic (All Races)	2	9.1%
Tumor subtype	HR+/HER2−	9	40.9%
	HR+/HER2+	5	22.7%
	HR−/HER2+	1	4.5%
	Triple negative	3	13.6%
	Unknown	4	18.2%
Grade	I	0	0%
	II	5	22.7%
	III/IV	11	50%
	Unknown	6	27.3%
Histology	Ductal	22	100%
	Lobular	0	0%
	Ductal and lobular	0	0%
	Other	0	0%
Bone metastases	No	6	27.3%
	Yes	16	72.7%
Liver metastases	No	17	77.3%
	Yes	4	18.2%
	Unknown	1	4.5%
Lung metastases	No	9	40.9%
	Yes	12	54.5%
	Unknown	1	4.5%
Distant lymph node metastases	No	5	22.7%
	Yes	1	4.5%
	Unknown	16	72.7%
Other metastases	No	5	22.7%
	Yes	2	9.1%
	Unknown	15	68.2%
Surgery (mastectomy or lumpectomy)	No	18	81.8%
	Yes	4	18.2%
Radiation	No or Unknown	9	40.9%
	Yes	13	59.1%
Chemotherapy	No or Unknown	6	27.3%
	Yes	16	72.7%
Marital status at diagnosis	Single	6	27.3%
	Married	12	54.5%
	Other (Separated/Divorced/Widowed)	3	13.6%
	Unknown	1	4.5%
Median household income	≥USD 75,000	8	36.4%
	USD 65,000–74,999	5	22.7%
	USD 55,000–64,999	5	22.7%
	USD 45,000–54,999	3	13.6%
	USD 35,000–44,999	1	4.5%

Abbreviations: NHAPI, Non-Hispanic Pacific Islander; HR, hormone receptor; HER, human epidermal growth factor receptor.

**Table 2:** Overall survival based on clinical and demographic characteristics.

Variable	Median OS (Months)	Log-Rank *p*
**Age at diagnosis**		
<50 years	9	<0.53
50–64 years	8	
>64 years	4	
**Grade**		
II	6	0.9
III/IV	10	
**Tumor subtype**		
HR+/Her2-	13	0.19
HR+/Her2+	6	
HR-/Her2+	-	
Triple negative	5	
Unknown	1	
**Race**		
NHW	9	0.59
NHB	5	
NHAPI	1	
Hispanic	8	
**Surgery**		
No	8	0.14
Yes	5	
**Radiation**		
No/Unknown	10	0.24
Yes	5	
**Chemotherapy**		
No/Unknown	3	0.004
Yes	10	
**Liver metastases**		
No	8	0.04
Yes	2	
**Lung metastases**		
No	6	0.65
Yes	8	
**Other metastases**		
No	5	0.71
Yes	8	
**Marital Status**		
Single	5	0.25
Married	8	
Other	4	

Abbreviations: OS, overall survival; CI, confidence interval; NHW, Non-Hispanic white; NHB, Non-Hispanic black; NHAPI; Non-Hispanic pacific islander; HR, hormone receptor; HER, human epidermal growth factor receptor.

## Data Availability

The data presented in this study are available on request from the corresponding author.
